# Development and validation of the Japanese version of the Bedtime Procrastination Scale (BPS-J)

**DOI:** 10.1186/s40359-024-01557-4

**Published:** 2024-02-01

**Authors:** Megumi Hazumi, Aoi Kawamura, Takuya Yoshiike, Kentaro Matsui, Shingo Kitamura, Ayumi Tsuru, Kentaro Nagao, Naoko Ayabe, Tomohiro Utsumi, Muneto Izuhara, Mio Shinozaki, Eriya Takahashi, Michio Fukumizu, Momo Fushimi, Satomi Okabe, Taisuke Eto, Daisuke Nishi, Kenichi Kuriyama

**Affiliations:** 1 Department of Public Mental Health Research, National Institute of Mental Health, National Center of Neurology and Psychiatry, Tokyo, Japan; 2 Department of Sleep-Wake Disorders, National Institute of Mental Health, National Center of Neurology and Psychiatry, Tokyo, Japan; 3 Department of Laboratory Medicine, National Center Hospital, National Center of Neurology and Psychiatry, Tokyo, Japan; 4 Department of Psychiatry, National Center Hospital, National Center of Neurology and Psychiatry, Tokyo, Japan; 5 Department of Regional Studies and Humanities, Faculty of Education and Human Studies, Akita University, Akita, Japan; 6 Department of Psychiatry, The Jikei University School of Medicine, Tokyo, Japan; 7 Segawa Memorial Neurological Clinic for Children, Tokyo, Japan; 8 Department of Mental Health, Graduate School of Medicine, The University of Tokyo, Tokyo, Japan

**Keywords:** Bedtime procrastination, Sleep loss, Chronotype, Questionnaire, Validation, Self-control, General procrastination

## Abstract

**Background:**

The average sleep duration of Japanese people is shorter than that of people from other countries, and bedtime procrastination is suspected to be one of the factors contributing to this issue. This study aimed to develop and validate the Japanese version of the Bedtime Procrastination Scale (BPS-J).

**Methods:**

The BPS-J was developed through procedures including the translation and back-translation of the scale, cognitive interviews with 100 participants who reported having experiences of being diagnosed with insufficient sleep syndrome (ISS) or receiving treatment for ISS using open-ended online questionnaires, and expert checking. To investigate the scale’s validity and reliability, an online survey was conducted with daytime workers aged 20 − 65 years without a history of sleep disorders other than ISS. Half the participants were retested using the same survey after 14 days. Participants’ responses to the Brief Self-Control Scale (BSCS), General Procrastination Scale (GPS), and Munich ChronoType Questionnaire (MCTQ), and data on sleep-related variables such as sleep duration on workdays and the days per week of fatigue or sleep loss, sex, and age, were collected.

**Results:**

We analyzed data from 574 participants to assess scale validity. We then analyzed data from 280 participants to determine test–retest reliability. Confirmatory factor analyses revealed that the two-factor model without Item 2 was most suitable for the BPS-J, unlike other language versions. Regardless of the full-item model or the model with Item 2 eliminated, sufficient reliability and significant correlations with the BSCS, GPS, MCTQ, and sleep-related variables such as sleep duration per night on work days, days per week of feeling fatigued, and days per week of sleep loss were observed. Logistic and linear regressions showed that the relationships between the BPS-J, sleep-related variables, and MCTQ were maintained after adjusting for sex and age.

**Conclusion:**

The BPS-J had sufficient validity and reliability. Further, eliminating Item 2 from the original version of the BPS strengthened the ability to survey Japanese daytime workers.

**Supplementary Information:**

The online version contains supplementary material available at 10.1186/s40359-024-01557-4.

## Background

Getting sufficient sleep at the appropriate time is important for maintaining physical and mental health and performance. It has been well established that chronic sleep deprivation or delay in the sleep phase increases the prevalence of diabetes, obesity, and cardiovascular diseases [1–3]. It has also been reported that various mental disorders, including major depression, are associated with sleep problems [3–5]. In addition, sleep loss interferes with cognitive function [6–9] and emotion regulation [10–13], resulting in difficulties in social adjustment [14]. Therefore, getting sufficient sleep at the appropriate time is essential not only for individuals’ health but also for socioeconomic benefits.

Bedtime procrastination, the behavioral tendency of staying up later at night than intended or needed to go to bed, is suggested to be one of the factors promoting shorter sleep duration [15, 16]. Several cross-sectional studies have indicated a relationship between bedtime procrastination and sleep loss. In the general population, individuals with bedtime procrastination tend to have insufficiently short sleep duration, especially on workdays [17]. Adolescents who procrastinated their bedtime tended to have short sleep durations, and those with evening chronotypes showed more severe sleep deprivation [18]. In addition, bedtime procrastination has been suggested to share a core concept of low self-control and general procrastination. Several studies have indicated that self-control and general procrastination are associated with bedtime procrastination [15, 19–22].

Among the Japanese, bedtime procrastination are suggested to results in a shorter sleep duration and being more prone to an evening chronotype than among non-Japanese individuals. Specifically, the average sleep duration of Japanese people is much shorter than that of people from 63 other countries [23]. Further, the evening chronotype is more prevalent in Japanese young adults than in young adults from other countries [24, 25].

To measure the tendency toward bedtime procrastination, the Bedtime Procrastination Scale (BPS) was developed and validated by Kroese et al. [15]. This self-reported outcome measurement consists of nine items measuring subjective bedtime procrastination. To our knowledge, it is the first scale developed to assess bedtime procrastination. The BPS is a major measurement tool translated and validated in several languages, such as Dutch, Spanish, English, and Korean [15, 19, 20, 22]. However, a Japanese version of the BPS has not yet been developed. The coronavirus disease 2019 (COVID-19) pandemic has particularly brought out the burdens of sleep loss and delayed sleep–wake phase on sleep health worldwide [26–29]. Bedtime procrastination has been regarded as a factor causing sleep problems during the COVID-19 pandemic [30–32]. In addition, the development of the Japanese version of the BPS may help explain the shorter sleep duration of Japanese people in comparison to people from other countries [23–25]. Therefore, this study aimed to develop and confirm the validity and reliability of the Japanese version of the Bedtime Procrastination Scale (BPS-J).

## Methods

### Translation and cross-cultural adaptation processes in the development of the BPS-J

After obtaining permission from the developer of the original version of the BPS, the BPS-J was developed according to the COSMIN reporting guideline in four steps: forward translation, back-translation, cognitive interviews using the questionnaire, and expert checking [33].

First, forward translation was performed by a sleep expert and a non-expert, both being native Japanese speakers. Then, two other sleep experts integrated the two translations by comparing and adopting the expressions.

Second, the BPS-J was back-translated by three non-experts who were bilingual in English and Japanese, unlike the non-expert translator mentioned above. After the BPS-J was back-translated by the first bilingual non-expert, the second bilingual non-expert independently cross-checked it, and the third bilingual non-expert performed the final confirmation. In instances where the back-translated English expressions were very different from the original version, two sleep experts who integrated the draft translations revised the forward-translated version, and the back-translation process was repeated. This process was repeated until the English expressions in the back-translated BPS-J completely matched those in the original BPS.

### Linguistic validation of the BPS-J

We conducted cognitive interviews with individuals with suspected insufficient sleep syndrome (ISS) for the linguistic validation of the BPS-J [34], as individuals with ISS were suspected to have bedtime procrastination tendency based on individuals with high BPS scores are reported to have less sleep [15]. A total of 100 participants (male = 53%, mean age = 43.56) who reported having experiences of being diagnosed with ISS or receiving treatment for ISS were recruited through an Internet survey agency (Rakuten Insight, Tokyo, Japan). They were asked to write their opinions about the comprehensibility, comprehensiveness, and relevance of the BPS-J in open-ended fields. The instruction statements were as follows: “*Did you find the instructional text and each question item on this scale easy to understand? Please indicate any instructional text or questions that were difficult to understand. If there are alternatives, please indicate them as well*”; “*Do you think this scale includes the experiences associated with your bedtime procrastination behaviors? If you think any important experiences or characteristics often associated with bedtime procrastination are missing, please describe them (if not, please do not fill in the blanks)*”; and “*Does each item on this scale correspond to your experiences with bedtime procrastination? If there are items that you think are not related to your bedtime procrastination behaviors, please indicate so (questions 2, 3, 7, and 9 ask about the behavior of those who do not procrastinate going to bed).*” Each statement was accompanied by the note “*If you think there is no particular problem, please leave this field blank.*”

The expressions or items in the BPS-J pointed out by two or more participants were revised based on discussion. To improve comprehension, Japanese translations of “*rarely*” and “*frequent*” were revised based on the suggestions by five participants. Revisions in terms of the comprehensiveness and relevance of the items were not made because there were no participant suggestions regarding points that they felt should be modified. Further details regarding the suggestions in terms of comprehensibility, relevance, and comprehensiveness are presented in Additional file 1: Appendix 1.

After three sleep experts performed these checks, they approved the final version of the BPS-J (Additional file 2: Appendix 2).

### Testing the validity, contribution, and reliability of the BPS-J

#### Participants

We recruited 600 individuals who reported meeting the following criteria: a) between the ages of 20 and 65 years, b) daytime worker (working four or more days a week), and c) no history of sleep disorders other than ISS. People whose waking times and social constraints on activities varied from day to day were excluded, in accordance with the definition of bedtime procrastination.

Among the participants who completed the questionnaire, those who met the following criteria based on the answers were excluded from the analyses: a) inconsistency between answers (e.g., overlap between sleeping and working time), and b) those who selected incorrect answers to the dummy question.

#### Survey design

All participants’ data were collected employing a cross-sectional design to estimate structural and criterion validities, internal consistency, measurement error, and relation of the BPS-J. To confirm the test–retest reliability, half of the participants were asked to answer the same questionnaire again after 14 days [35]. All participants were asked to complete the test–retest reliability questionnaire on a first-come, first-served basis, and data collection was stopped when half of the participants had completed the questionnaire.

Online surveys were conducted through Rakuten Insight, Inc., one of the largest online research service companies in Japan. Participants were awarded points by the company after completing the entire questionnaire; the points could be used for shopping and other purposes. Once candidates received a request to participate in the survey, they voluntarily accessed the online page of the survey system. If they agreed to participate after receiving informed consent, they answered screening questions to ensure that they met the inclusion criteria for study participants. When they met the criteria, they completed the entire questionnaire.

#### Measurements

##### BPS-J

The BPS measures the tendency of bedtime procrastination, which has been confirmed to have sufficient validity and reliability in the original and other language versions [15, 17, 20, 22]. This tool consists of nine items, and each item is evaluated on a 5-point scale: 1 (*almost never*) to 5 (*almost always*). The BPS total score can be calculated in two ways: by calculating the average of the items [15, 17] or by simply summing them [20, 22]. Higher total scores indicate a greater tendency of bedtime procrastination. The values for the alternatives to inverted items, such as Items 2, 3, 7, and 9, were reversed before calculation.

##### Brief self-control scale

The Brief Self-Control Scale (BSCS) [36] measures the degree of self-control, which is the ability to control one’s own emotions and desires to achieve more important long-term benefits [37, 38], and consists of 13 items. Each item is evaluated from 1 (*not at all like me*) to 5 (*just like me*), and higher scores indicate greater difficulty in self-control. Validity and reliability were confirmed in both the original [36] and the Japanese versions [39].

##### General procrastination scale

The General Procrastination Scale (GPS) [40] measures the tendency of general procrastination, which is the most common and ubiquitous behavioral pattern of laying off things that need to be done in all aspects of life [41, 42]. Validity and reliability were confirmed for both the original [40] and the Japanese [43] versions. The Japanese version of this scale consists of 13 items [43], and each item is evaluated on a scale from 1 (*never*) to 5 (*almost always*). A higher total score indicates a stronger tendency for procrastination.

##### Sleep-related variables

Data on the sleep and related subjective variables were collected based on the items used in the study by Kroese et al. (2014) [15]. These items were generated originally by Kroese et al. [15], and items directly translated from the original were used in this study. Therefore, these items do not have established validity or reliability. Thus, we also adopted the Munich ChronoType Questionnaire (MCTQ) to clarify the relationship between the BPS-J and sleep–wake phase delay.

Sleep duration on workdays was measured using the item “*How long is your sleep duration on workdays?*” and evaluated with the following alternatives: less than 5 h, less than 6 h, less than 7 h, less than 8 h, less than 9 h, less than 10 h, and 10 h or over. Number of days per week feeling fatigued during the daytime was measured with the item “*How many days do you experience fatigue per week?*” Number of days per week of feeling sleep loss was measured with the item, “*How many days do you experience insufficient sleep per week?*” The answers to these two questions were categorized as 0 days, 1 − 2 days, 3 − 4 days, 5 − 6 days, and 7 days. The tendency to view bedtime procrastination as a problem was measured with the item “*To what extent do you find it problematic that you go to bed later than you would like to?*” on a scale of 1 (not at all) to 5 (very much).

##### Munich chronotype questionnaire

MCTQ was used to measure participant chronotype by assessing the midpoint of the sleep in work-free days correcting sleep loss. Although the MCTQ has not been examined in previous validation studies of the BPS, it was adopted in this study to understand the relationship between the BPS-J and sleep–wake phase delay.

The MCTQ measures the propensity to sleep at a particular time during a 24-h period, which is potentially regulated by individual circadian rhythmicity. Validity and reliability were confirmed in the original and the Japanese versions [44, 45]. Among the 17 items regarding sleep habits, the following items on workdays and work-free days were used: local time of going to bed, local time of preparing to sleep, sleep latency, and sleep end. With these items, several variables were calculated in line with validation studies [44, 45]. Sleep duration (the interval of sleep onset [local time of preparing to sleep + sleep latency] and sleep end) and midsleep (time [sleep duration ÷ 2] minutes elapsed after sleep onset) on workdays and work-free days as well as average sleep duration per week ([sleep duration on workdays × workdays per week + sleep duration on work-free days × {7-workdays per week}]/7) were calculated. Furthermore, absolute social jetlag (midsleep on weekdays—midsleep on work-free days) and weekly sleep loss ( [average sleep duration per week—sleep duration on workdays] × workdays per week; [average sleep duration per week—sleep duration on work-free days] × [7—workdays per week]) were calculated. Additionally, absolute social jetlag was divided into groups of < 120 min and ≥ 120 min, based on the cut-off point of the existing studies [46–48]. Work start time and work end time, which are parts of the seven items in the domain of work details, were also used for calculating working hours.

#### Statistical analyses

The results of the analyses were defined as statistically significant when the *p*-value was < 0.05. Analyses of criterion validity were performed using SPSS AMOS version 25 (IBM Japan, Tokyo), and the other analyses were performed using SPSS Statistics version 28 (IBM Japan, Tokyo). Missing values were handled by multiple imputation.

### Participants’ characteristics and each item of the BPS-J

The median and Interquartile range (IQR) for continuous variables and the number and proportion for categorical variables were calculated.

To examine whether conditions have changed between Time 1 (T1) and Time 2 (T2), Friedman’s tests for continuous variables and McNemar’s tests for categorical variables were performed using data from individuals who participated in both T1 and T2. The mean, SD, median, and interquartile range (IQR) of each BPS-J item at T1 were also calculated.

#### Structural validity

Confirmatory factor analysis (CFA) for the one-factor model [15, 17, 20, 22] was performed after the Kaiser–Meyer–Olkin (KMO) test and Bartlett’s test of Sphericity. For goodness of fit, *χ2/df*, the Tucker-Lewis index (TLI), comparative fit index (CFI), and root mean square error of approximation (RMSEA) were calculated. We determined the model to be good when TLI, CFI, and RMSEA were > 0.90, > 0.90, and < 0.06, respectively [49]. When the model did not meet these criteria, we eliminated items with low factor loadings (< 0.50) and analyzed the model again [50].

Additionally, exploratory factor analysis (EFA) with maximum-likelihood and Promax rotation was performed to confirm whether the one-factor model was appropriate for the BPS-J, and CFA was performed again based on the results of the EFA. The appropriate number of factors was determined by the factor number with eigenvalues greater than 1 in the EFA scree plot. The mean, SD, median, and IQR of the total BPS-J scores were calculated based on the structural validity results. These analyses were performed on the sum and average scores.

#### Criterion validity

Bivariate Spearman’s correlation analyses between the BPS-J total score and the following variables were performed: age, BSCS, GPS, sleep duration on workdays, days of feeling fatigue during daytime, days of feeling sleep loss during daytime, degree of viewing bedtime procrastination as a problem, and variables of MCTQ such as sleep duration on workdays, sleep duration on work-free days, midsleep on workdays, midsleep on work-free days, absolute social jetlag, absolute social jetlag ≥ 120 min, weekly sleep loss, working time, work start time, and work end time.

#### Reliability

Reliability was assessed using data from individuals who participated in both T1 and T2 in three ways: internal consistency, measurement error, and test–retest reliability. The internal consistency of each item and the BPS-J total score were confirmed using Cronbach’s α, MacDonald’s ω, and item total (I-T) correlation. The measurement error was confirmed by calculating the standard error of measurement (SEM) and the smallest detectable change (SDC) of the total score [51, 52]. Test–retest reliability was confirmed using intraclass correlation coefficient (ICC) and Pearson’s correlation coefficient between T1 and T2.

#### Relation of the BPS-J to sleep-related variables

To examine the association of the BPS-J to sleep loss, sleep–wake phase delay, daytime fatigue, and view of bedtime procrastination as a problem, with reference to prior literature [15], the linear regressions for continuous outcomes and logistic regression for categorical outcomes were performed. The MCTQ and sleep-related variables [15] were used as the dependent variables. The GPS and BSCS, which were suggested to be associated with both the BPS and sleep-related variables in a previous study [15], were also included in the analysis models as covariates in addition to demographic information such as sex and age.

## Results

### Participants’ characteristics and the BPS-J

**Fig. 1:**
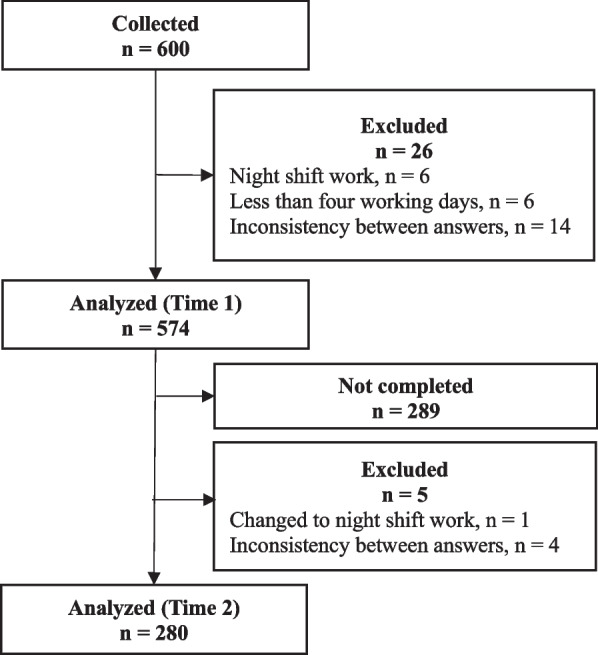
Flow chart of the participant selection process

The participant selection process is shown in Fig. 1. Out of 600 participants, data from 574 participants were analyzed to estimate the structural and criterion validities, relation of the BPS-J to sleep-related variables, internal consistency, and SEM and SDC of the BPS-J. Among them, data from 280 participants were retested and analyzed to estimate the test–retest reliability of the BPS-J. No missing values were observed.

Table 1 shows the demographic characteristics of the participants at T1 and T2. At T1, the mean age was 44.25 (SD = 12.84), and the proportion of males was 50%. Mean sleep duration on workdays and work-free days were 393.09 (SD = 69.33) min and 440.89 (SD = 93.10) min, respectively. At T2, the mean age was 44.34 (SD = 12.74), and the proportion of males was 51.4%. Mean sleep duration on workdays and work-free days were 394.50 (SD = 67.84) min and 443.11 (SD = 72.46) min, respectively. The characteristics of the participants at T1 and T2 were comparable, except in terms of the Self-Control Scale, the number of feeling fatigue, and the number of days feeling sleep loss.

**Table 1:** Participants’ characteristics

	Time 1 *n* = 574		Time 2 *n* = 280			
	Median or n	SD or %	Median or n	SD or %		
Sex						
Male	287	50.00%	144	51.40%		
Female	287	50.00%	136	48.60%		
Age (years)	45.00	33.00 – 55.00	45.00	33.00 – 55.00		
Self-control scale	36.00	30.00 – 41.00	37.00	30.00 – 41.00	*χ*^*2*^ = 5.69	***p* = 0.02**
General procrastination scale	34.00	26.75 – 39.00	34.00	25.50 –39.50	*χ*^*2*^ = 1.56	*p* = 0.21
Feeling fatigue (days per week)	2.00	0.00 – 3.00	1.00	0 – 3	*χ*^*2*^ = 8.03	***p* = 0.005**
0 days	168	29.30%	86	30.70%	*z* =—0.23	*p* = 0.82
1 – 2 days	229	39.90%	107	38.20%		229
3 – 4 days	92	16.0%	40	14.30%		
5 – 6 days	54	9.40%	30	10.70%		
7 days	31	5.40%	17	6.10%		
Feeling sleep loss (days per week)	2.00	0.00 – 3.00	2.00	0 – 2	*χ*^*2*^ = 0.63	***p* < 0.001**
0 days	168	29.30%	81	28.90%	*z* =—3.27	***p* < 0.001**
1 – 2 days	196	34.10%	87	31.10%		
3 – 4 days	103	17.90%	51	18.20%		
5 – 6 days	75	13.10%	44	15.70%		
7 days	32	5.60%	17	6.10%		
Viewing bedtime procrastination as a problem						
1 not at all	86	15.00%	40	14.30%	*z* =—0.36	*p* = 0.72
2	100	17.40%	52	18.60%		
3	165	28.70%	88	31.40%		
4	175	30.50%	75	26.80%		
5 very much	48	8.40%	25	8.90%		
Sleep duration on workdays						
Less than 5 h	35	6.10%	15	5.40%	*z* =—0.002	*p* = 0.99
Less than 6 h	570	34.00%	94	33.60%		
Less than 7 h	210	36.60%	106	37.90%		
Less than 8 h	108	18.80%	50	17.90%		
Less than 9 h	22	3.80%	11	3.90%		
Less than 10 h	3	0.50%	2	0.70%		
10 h and over	1	0.20%	2	0.70%		
Munich ChronoType Questionnaire						
Sleep duration on workdays	390.00	350.00 – 440.00	398.50	355.00 – 440.75	*χ*^*2*^ = 1.83	*p* = 0.18
Sleep duration on work-free days	445.00	390.00 – 495.00	445.00	385.00 – 497.00	*χ*^*2*^ = 1.28	*p* = 0.26
Midsleep on workdays	3:00	2:22 – 3:38	3:02	2:22 – 3:45	*χ*^*2*^ = 0.07	*p* = 0.80
Midsleep on work-free days	3:35	2:50 – 4:32	3:39	2:47 – 4:35	*χ*^*2*^ = 0.69	*p* = 0.41
Absolute social jetlag (minutes)	35.00	7.00 – 67.00	30.00	4.25 – 62.00	*χ*^*2*^ = 0.004	*p* = 0.95
Absolute social jetlag ≥ 120 min	51	8.90%	19	6.80%	*z* =—0.23	*p* = 0.82
Weekly sleep loss (minutes)	42.00	0.00 – 120.00	42.00	0.00 – 128.00	*χ*^*2*^ = 0.63	*p* = 0.43
Work start time	8:40	8:15 – 9:00	8:45	8:25 – 9:00	*χ*^*2*^ = 0.06	*p* = 0.81
Work end time	17:30	17:00 – 18:30	17:40	17:00 – 18:30	*χ*^*2*^ = 0.17	*p* = 0.68
Working time (minutes)	540.00	540.00 – 570.00	540.0	510.0 0– 582.50	*χ*^*2*^ = 0.22	*p* = 0.64

*SD* standard deviation, *t* t value of paired t-test, *z* z value of McNemar test; Bold = *p* < 0.05

Table 2 shows the mean, SD, median, and IQR for each item of the BPS-J.

**Table 2:** Item characteristics of the BPS-J

	**Mean**	**SD**	**Median**	**IQR**
**Item 1**	2.79	± 1.12	3	2–4
**Item 2**	3.37	± 1.09	3	3–4
**Item 3**	3.39	± 1.32	3	2–5
**Item 4**	2.77	± 1.18	3	2–4
**Item 5**	2.45	± 1.15	2	2–3
**Item 6**	2.57	± 1.17	2	2–3
**Item 7**	3.05	± 1.30	3	2–4
**Item 8**	2.6	± 1.19	3	2–3
**Item 9**	3.07	± 1.23	3	2–4

*SD* standard deviation, *IQR* interquartile range

Flowchart of the selection process of participants from participant recruitment to data analysis at Time 1 and Time 2.

#### Structural validity

Table 3 shows the results of the CFA. As the result of the KMO test was 0.86 (*p* < 0.001) and the result of Bartlett’s test was significant (χ^2^ = 2221.26, df = 28, *p* < 0.001), CFA was interpreted as appropriate. The values of the factor loading and fit indices of the one-factor model are listed in Table 3. Although the factor loadings for all items were *p* < 0.001, the fit indices were poor (χ^2^/df = 15.55, *p* < 0.001, TLI = 0.77, CFI = 0.83, RMSEA = 0.16). The factor loadings of Item 2, “*I go to bed early if I have to get up early in the morning (invert scale)*,” and Item 3, “*If it is time to turn off the lights at night I do it immediately (invert scale)*,” were under 0.50. Therefore, based on the value of factor loading, CFAs of the models with Item 2 eliminated (KMO = 0.86, *p* < 0.001; χ^2^ = 2221.26, df = 28, *p* < 0.001) and with Items 2 and 3 eliminated (KMO = 0.85, *p* < 0.001; χ^2^ = 2041.16, df = 21, *p* < 0.001) were additionally performed. The fit indices of the model with Item 2 eliminated were slightly better than those of the full-item model, except for the RMSEA (χ2/df = 18.92, *p* < 0.001, TLI = 0.77, CFI = 0.84, RMSEA = 0.18).

**Table 3:** Confirmatory factor analyses results of the BPS-J

	**KMO**	**One-factor model**			**Two-factor model**			
		Full items	Item 2 eliminated	Items 2 & 3 eliminated	Full Items		Item 2 eliminated	
Item 1	0.88	0.70	0.71	0.72	0.56	–	0.76	–
Item 2	0.81	- 0.19	–	–	–	0.23	–	–
Item 3	0.87	- 0.45	- 0.45	–	–	0.51	–	0.50
Item 4	0.85	0.77	0.77	0.78	0.83	–	0.83	–
Item 5	0.89	0.72	0.72	0.73	0.77	–	0.77	–
Item 6	0.83	0.78	0.78	0.77	–	- 0.83	–	- 0.83
Item 7	0.82	- 0.71	- 0.71	- 0.69	–	0.84	–	0.84
Item 8	0.90	0.77	0.77	0.77	0.75	–	0.75	–
Item 9	0.86	- 0.58	- 0.57	- 0.55	–	- 0.61	–	- 0.6
*χ2/df*		15.55	18.92	21.90	9.10		10.70	
*P*		< 0.001	< 0.001	< 0.001	< 0.001		< 0.001	
*TLI*		0.77	0.77	0.78	0.87		0.88	
*CFI*		0.83	0.84	0.86	0.91		0.92	
*RMSEA*		0.16	0.18	0.19	0.12		0.13	

*KMO* Kaiser–Meyer–Olkin, *p* probability value, χ2 chi-square, *df* degrees of freedom, *TLI* Tucker–Lewis index, *CFI* Comparative Fit Index, *RMSEA* Root Mean Square of Approximation, *Full items* the full-item model, *Item 2 eliminated* the Item 2 eliminated model, *Items 2 and 3 eliminated* the Items 2 and 3 eliminated model

**Fig. 2:**
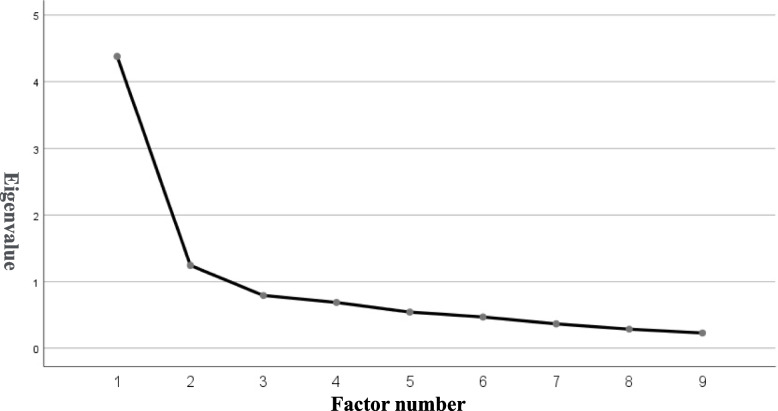
Scree plot of the exploratory factor analysis

As other models were suggested to be appropriate from the fit indices of the one-factor model, an EFA was performed. The eigenvalues of the EFA showed that the two-factor model was appropriate (Fig. 2). CFAs of the two-factor model were performed (KMO = 0.86, *p* < 0.001; χ^2^ = 2279.04, df = 36, *p* < 0.001). Although the two-factor full-item model was better than the one-factor model (χ2/df = 9.10, *p* < 0.001, TLI = 0.87, CFI = 0.91, RMSEA = 0.12), Item 2 was eliminated and confirmed again because of low factor loading (KMO = 0.86, *p* < 0.001; χ^2^ = 2221.26, df = 28, *p* < 0.001; χ2/df = 10.70, *p* < 0.001, TLI = 0.88, CFI = 0.92, RMSEA = 0.13). Factor 1 and 2 were interpreted as “Preparing for bedtime” and “Adherence to bedtime”, respectively.

Based on the results of the CFA and EFA, the mean of mean score and mean of sum score of the BPS-J for the models with all the items, with Item 2 eliminated, and with Items 2 and 3 eliminated were calculated (Table 4). The mean sum scores were 24.32 (SD = 7.36), 21.69 (SD = 7.05), and 19.07 (SD = 6.36), and the median values were 25 (IQR = 19 – 29), 22 (IQR = 16 – 26), and 19 (IQR = 14 – 23), respectively. The mean average scores were 2.71 (SD = 0.81), 2.72 (SD = 0.87), 2.74 (SD = 0.91), and the median values were 2.78 (IQR = 2.11—3.22), 2.75 (IQR = 2.0 – 3.25), and 2.79 (IQR = 2.0 -3.29), respectively.

**Table 4:** Characteristics and reliability of the BPS-J total score

	**Full items**	**Item 2 eliminated**	**Item2 & 3 eliminated**
**Average score**			
Mean	2.71	2.72	2.74
SD	0.81	0.87	0.91
Median	2.78	2.75	2.79
IQR	2.11 – 3.22	2.0 – 3.25	2.0 – 3.29
ICC	0.78	0.78	0.78
Pearson's r	0.78	0.78	0.78
SEM	0.38	0.41	0.42
MDC	1.06	1.13	1.18
**Sum score**			
Mean	24.32	21.69	19.07
SD	± 7.36	± 7.05	± 6.36
Median	25	22	19
IQR	19 – 29	16 – 26	14 – 23
ICC	0.77	0.78	0.78
Pearson's r	0.78	0.78	0.78
SEM	3.51	3.28	2.98
SDC	9.74	9.08	8.28
**Cronbach's α**	0.86	0.87	0.88
**McDonald's ω**	0.86	0.87	0.88

*SD* standard deviation, *IQR* interquartile range, *ICC* interclass correlation coefficient, *SEM* standard error of measurement, *SDC* smallest detectable change, *Full items* the full-item model, *Item 2 eliminated* the Item 2 eliminated model, *Items 2 and 3 eliminated* the Items 2 and 3 eliminated model

The scree plot of the exploratory factor analysis shows that only the eigenvalues of Factors 1 and 2 are greater than 1. Thus, the two-factor model is considered to be appropriate for the Japanese version of the Bedtime Procrastination Scale (BPS-J).

#### Criterion validity

Table 5 shows the bivariate correlation between the BPS-J and the variables. Regardless of the model, significant correlations between the BPS-J and the variables were shown, except for the degree of viewing bedtime procrastination as a problem, working time, and work start time.

**Table 5:** Bivariate correlation between the BPS-J and variables

	Full item		Item 2 Eliminated		Item2 & 3 Eliminated	
	*Spearman's ρ*	*p*	*Spearman's ρ*	*p*	*Spearman's ρ*	*p*
Brief self-control scale	0.40	** < 0.001**	0.40	** < 0.001**	0.41	** < 0.001**
General procrastination scale	0.49	** < 0.001**	0.48	** < 0.001**	0.49	** < 0.001**
Sleep duration on workdays	- 0.35	** < 0.001**	- 0.32	** < 0.001**	- 0.31	** < 0.001**
Feeling fatigue at daytime	0.33	** < 0.001**	0.33	** < 0.001**	0.34	** < 0.001**
Feeling sleep loss at daytime	0.48	** < 0.001**	0.48	** < 0.001**	0.50	** < 0.001**
Viewing bedtime procrastination as a problem	- 0.02	0.68	0.01	0.68	0.03	0.84
Munich ChronoType Questionnaire						
Sleep duration on workdays	- 0.33	** < 0.001**	- 0.34	** < 0.001**	- 0.33	** < 0.001**
Sleep duration on work-free days	- 0.13	**0.001**	- 0.12	**0.004**	- 0.10	**0.02**
Midsleep at workdays	0.40	** < 0.001**	0.41	** < 0.001**	0.41	** < 0.001**
Midsleep at work-free days	0.42	** < 0.001**	0.43	** < 0.001**	0.44	** < 0.001**
Absolute social jetlag	0.21	** < 0.001**	0.22	** < 0.001**	0.24	** < 0.001**
Absolute social jetlag ≥ 120 min	0.13	**0.002**	0.16	**0.007**	0.17	**0.004**
Weekly sleep loss	0.14	** < 0.001**	0.14	** < 0.001**	0.16	** < 0.001**
Working time	0.07	0.07	0.08	0.052	0.07	0.08
Work start time	0.10	0.07	0.07	0.07	0.08	0.08
Work end time	0.11	**0.009**	0.12	**0.004**	0.12	**0.004**

*P* probability value, *Full items* the full-item model, *Item 2 eliminated* the Item 2 eliminated model, *Items 2 and 3 eliminated* the Items 2 and 3 eliminated model; Bold = *p* < .05

#### Reliability

As presented in Table 4, Cronbach’s α was 0.86, 0.87, and 0.88, and McDonald's ω was 0.86, 0.87, and 0.88, respectively. Table 6 shows the I-T correlation, Cronbach’s α and McDonald's ω when each item is eliminated. The average I-T correlations of the models with all the items, with Item 2 eliminated, and with Items 2 and 3 eliminated were 0.58, 0.73, and 0.76, respectively. Cronbach’s α and McDonald's ω of factor 1, factor 2 with all items, factor 2 with Item 2 eliminated were 0.86 and 0.86, 0.76 and 76, and 0.79 and 0.79.

**Table 6:** I-T correlation, Cronbach’s α, and McDonald’s ω when each item is eliminated

	**ICC of each item**	**Full item**			**Item 2 eliminated**			**Item2 & 3 eliminated**		
		I-T correlation	α when each item eliminated	ω when each item eliminated	I-T correlation	α when each item eliminated	ω when each item eliminated	I-T correlation	α when each item eliminated	ω when each item eliminated
**Item 1**	0.59	0.60	0.84	0.84	0.71	0.86	0.86	0.74	0.87	0.87
**Item 2**	0.55	- 0.21	0.87	0.87	–	–	–	–	–	–
**Item 3**	0.46	- 0.47	0.85	0.86	- 0.58	0.88	0.88	–	–	–
**Item 4**	0.59	0.67	0.83	0.84	0.78	0.85	0.85	0.80	0.86	0.86
**Item 5**	0.57	0.64	0.84	0.84	0.74	0.86	0.85	0.76	0.86	0.86
**Item 6**	0.68	0.70	0.83	0.83	0.80	0.85	0.85	0.81	0.86	0.86
**Item 7**	0.62	- 0.68	0.83	0.83	- 0.76	0.85	0.85	- 0.75	0.86	0.86
**Item 8**	0.56	0.69	0.83	0.83	0.78	0.85	0.85	0.81	0.86	0.86
**Item 9**	0.49	- 0.58	0.84	0.85	- 0.66	0.87	0.86	- 0.63	0.88	0.88

*ICC* interclass correlation coefficient, *I-T correlation* item total correlation, *α* Cronbach’s α, *ω* McDonald’s ω, *Full items* the full-item model, *Item 2 eliminated* the Item 2 eliminated model, *Items 2 and 3 eliminated* the Items 2 and 3 eliminated model

Table 4 shows the SEM and SDC of the models with all the items, with Item 2 eliminated, and with Items 2 and 3 eliminated. The SEM values of the average score were 0.38, 0.41, and 0.42, and those of the MDC were 1.06, 1.13, and 1.18, respectively. The SEM values of the sum score were 3.51, 3.28, and 2.98, and those of the MDC were 9.74, 9.08, and 8.28, respectively.

As Table 4 shows, ICC and Pearson’s correlation were almost comparable between the versions (ICC = 0.77, 0.77, and 0.78; r = 0.78, 0.78, and 0.78, respectively). Each item of ICC was between 0.46 and 0.68 (Table 6).

### Relation of the BPS-J to sleep-related variable

Table 7 shows the results of the multivariate linear and logistic regressions. BPS-J score was significantly related to the sleep-related variables defined by Kroese et al. [15, 19]: shorter sleep duration on workdays, more frequent days of feeling daytime fatigue, more frequent days of feeling sleep loss, and viewing bedtime procrastination as a problem less seriously.

**Table 7:** Results of the regression analyses between the BPS-J sum score and sleep-related variables

	Total		Item2 eliminated		Item2&3 eliminated	
Dependent variable		*p*		*p*		*p*
Sleep duration on workdays						
Less than 5 h	reference		reference		reference	
Less than 6 h	OR = 0.89	** < 0.001**	OR = 0.89	** < 0.001**	OR = 0.88	**0.001**
Less than 7 h	OR = 0.81	** < 0.001**	OR = 0.81	** < 0.001**	OR = 0.79	** < 0.001**
Less than 8 h	OR = 0.78	** < 0.001**	OR = 0.77	** < 0.001**	OR = 0.75	** < 0.001**
Less than 9 h	OR = 0.88	** < 0.001**	OR = 0.76	** < 0.001**	OR = 0.76	**0.002**
Less than 10 h	OR = 0.68	** < 0.001**	OR = 0.67	** < 0.001**	OR = 0.67	**0.002**
10 h and over	OR = 0.8	0.25	OR = 0.73	0.1	OR = 0.70	0.09
Feeling fatigue at daytime						
0 days	reference		reference		reference	
1 – 2 days	OR = 1.02	0.24	OR = 1.02	0.19	OR = 1.03	0.1
3 – 4 days	OR = 1.07	** < 0.001**	OR = 1.08	**0.002**	OR = 1.10	** < 0.001**
5 – 6 days	OR = 1.07	**0.01**	OR = 1.07	**0.02**	OR = 1.09	**0.008**
7 days	OR = 1.14	** < 0.001**	OR = 1.14	** < 0.001**	OR = 1.17	** < 0.001**
Feeling sleep loss at daytime						
0 days	reference		reference		reference	
1 – 2 days	OR = 1.09	** < 0.001**	OR = 1.1	** < 0.001**	OR = 1.13	** < 0.001**
3 – 4 days	OR = 1.17	** < 0.001**	OR = 1.17	** < 0.001**	OR = 1.21	** < 0.001**
5 – 6 days	OR = 1.23	** < 0.001**	OR = 1.24	** < 0.001**	OR = 1.29	** < 0.001**
7 days	OR = 1.31	** < 0.001**	OR = 1.32	** < 0.001**	OR = 1.38	** < 0.001**
Viewing bedtime procrastination as a problem						
1 not at all	reference		reference		reference	
2	OR = 0.96	0.12	OR = 0.97	0.22	OR = 0.97	0.29
3	OR = 0.96	**0.04**	OR = 0.96	0.07	OR = 0.96	0.15
4	OR = 0.96	**0.048**	OR = 0.97	0.18	OR = 0.98	0.4
5 very much	OR = 0.91	** < 0.001**	**OR = 0.92**	**0.007**	OR = 0.93	**0.03**
Munich ChronoType Questionnaire						
Sleep duration on workdays	β =—0.43	** < 0.001**	β =—0.42	** < 0.001**	β =—0.42	** < 0.001**
Sleep duration on work-free days	β =—0.21	** < 0.001**	β =—0.21	** < 0.001**	β =—0.18	** < 0.001**
Midsleep at workdays	β = 0.38	** < 0.001**	β = 0.38	** < 0.001**	β = 0.38	** < 0.001**
Midsleep at work-free days	β = 0.34	** < 0.001**	β = 0.34	** < 0.001**	β = 0.34	** < 0.001**
Absolute social jetlag	β = 0.06	0.26	β = 0.06	0.25	β = 0.08	0.10
< 120 min	reference		reference		reference	
≥ 120 min	OR = 1.02	0.5	OR = 1.02	0.41	OR = 1.04	0.21
Weekly sleep loss	β = 0.11	**0.03**	β = 0.10	**0.03**	β = 0.12	**0.01**

*P* probability value, *OR* odds ratio, *β* standardized regression coefficient, *Full items* the full-item model, *Item 2 eliminated* the Item 2 eliminated model, *Items 2 and 3 eliminated* the Items 2 and 3 eliminated model, *Bold p* < 0.05

Significant relationships between the BPS-J and MCTQ variables were also found: shorter sleep duration on workdays, shorter sleep duration on work-free days, delayed midsleep on workdays, delayed midsleep on work-free days, and weekly sleep loss. There was no significant relationship between the BPS-J and absolute social jetlag.

## Discussion

Except for structural validity, we confirmed that the BPS-J has acceptable validity and reliability, similar to other language versions. The two-factor version that eliminated Item 2 indicated slightly better validity than that of the full-item version. The factor loading of Items 2 and 3, especially Item 2, was not as high as that of other items, resulting in our consideration of eliminating Item 2 from the BPS-J scale. Behavioral patterns such as “promptly turning off the lights when getting up early the next morning” were suspected to be related to factors other than bedtime procrastination.

The structure of the two-factor model without Item 2 was better than that of the one-factor model or the full-item model for the BPS-J. The fit indices seemed to be lower than those of the other language versions, even in the two-factor model, in which the factor loading of items was eliminated. The factor loadings of Item 2 in the Korean version also seemed to be lower than those of the other items [20], similar to the trend noted for the BPS-J. Considering the fact that sleep duration among Japanese and Korean participants seemed to be shorter than those from other countries in the world [53], the trend implied by the poor fitting profile of Item 2 could be influenced by a mutually shared value of under-evaluation regarding getting sufficient sleep among societies with extremely short sleep duration^40^. The fact that the two-factor model after removing Item 2 fitted better than the one-factor model may not be peculiar to the BPS-J. The eigenvalues of the two components also reached 1 in the Polish and Korean versions, [17, 20] and the fit indices of the two-factor model were also better than the one-factor model for the Korean version^20^ as well as the BPS-J.

The mean total BPS-J score was almost the same as that of the original version. However, it seemed to be lower than that in the other language versions. Differences in the characteristics of the study participants could have caused discrepancies in the BPS scores. The participants of studies examining the BPS-J and BPS original versions were daytime workers [15], whereas those of the other language versions were students [17, 20, 22]. Considering the social constraint effect on bedtime procrastination [54], daytime workers may undertake more severe social responsibility than students [15, 17, 20, 22].

The criterion validity was consistent with other language versions of the BPS, considering that the BPS-J is associated with general procrastination, self-control, and sleep variables.

The reliability of the BPS-J was found to be sufficient, based on the Cronbach's α [55], McDonald's ω, ICC, [56] and correlation [57] between T1 and T2. The level of Cronbach's α was similar to that of the other language versions [17, 19, 20, 22, 58], but lower than that of the original version [15]. McDonald's ω, SEM, and MDC, which had previously only been calculated in the Polish version, were almost the same as those reported in the Polish version [17]. The correlation between T1 and T2, which was calculated in the Polish and Spanish versions, appears to be almost comparable to the Spanish version [22] and higher than the Polish version [17]. The correlation coefficient in the BPS-J was higher than that in the Polish version, possibly because the interval between T1 and T2 in the BPS-J was shorter than that in the Polish version [17].

The BPS-J is believed to enhance the assessment of underlying factors that contribute to ISS among daytime workers in Japan. This facilitates the implementation of interventions specifically targeting BP for those with ISS. This approach may be more effective than often ineffective traditional advice of simply telling individuals to go to bed earlier [59]. Such an approach allows for the implementation of specific behavioral interventions aimed at curbing BP by behavior replacement [60], thereby potentially reducing the prevalence of ISS.

This study has some limitations. The validity of the ISS diagnosis is subject to potential inaccuracies as it relies solely on participants’ self-reported accounts, although we devised a way to exclude dishonest respondents by eliminating those who responded inappropriately to the dummy question. Participants were limited to daytime workers, similar to the original version of the BPS. The BPS-J should be applied to other populations (e.g., students) with caution, based on the difference noted in the results of the BPS-J with daytime workers and other language versions of the BPS with students [20, 22]. Individuals whose bedtime procrastination tendency was so severe that they could not perform daytime work were not included in this study. Additional validation studies including such individuals are necessary for further clarification.

Regression analyses indicated that the BPS-J was associated with sleep-related variables, as with other language versions [15, 17, 20, 22]. The degree of “viewing bedtime procrastination as a problem,” the dependent variable used in Kroese et al.’s study [19], was similar to that used in a previous study [19]. This relationship may explain why patients with ISS hardly changed their sleep habits despite advice from sleep professionals [59]. Therefore, in the context of ISS, awareness of bedtime procrastination problems should be increased among people with the condition. Bedtime procrastination was also related to a delayed sleep–wake phase tendency and short sleep duration. These relationships are consistent with those reported in previous studies [61].

## Conclusion

We developed and validated the BPS-J, and constructed a two-factor model with Item 2 eliminated; the version with Item 2 eliminated was found to be more suitable for Japanese daytime workers. This study sheds light on the relationship between bedtime procrastination and sleep duration in Japanese populations, while also highlighting differences in bedtime procrastination tendencies between other countries and Japan. High BPS-J scores were indicative of increased harmful sleep-related variables in Japanese daytime workers, although the causal relationship remained unclear given the cross-sectional study design. Further studies are needed to clarify the causal relationships and degree of necessity of changing bedtime procrastination.

### Supplementary Information

**Additional file 1: Appendix 1.** Suggestions about comprehensibility, relevance, and comprehensiveness.

**Additional file 2: Appendix 2.** Translation of Instructions and items of the Bedtime Procrastination Scale.

## Data Availability

The data will be made available by the corresponding author upon request.

## Abbreviations

BPS-J: Japanese Version of the Bedtime Procrastination Scale
BSCS: Brief Self-Control Scale
CFA: Confirmatory Factor Analysis
CFI: Comparative Fit Index
COVID-19: Coronavirus Disease 2019
EFA: Exploratory Factor Analysis
GPS: General Procrastination Scale
ICC: Interclass Correlation Coefficient
IQR: Interquartile Range
ISS: Insufficient Sleep Syndrome
I-T: Item Total
KMO: Kaiser–Meyer–Olkin
MCTQ: Munich ChronoType Questionnaire
RMSEA: Root Mean Square Error of Approximation
SD: Standard Deviation
SDC: Smallest Detectable Change
SEM: Standard Error of Measurement
T1: Time 1
T2: Time 2
TLI: Tucker–Lewis index
