# Supplementary material for: Development and validation of the Japanese version of the Bedtime Procrastination Scale (BPS-J)
Source: BMC Psychol. 2024 Feb 1;12:56. doi: 10.1186/s40359-024-01557-4 (PMC10832274; doi:10.1186/s40359-024-01557-4)
Supplement: Supplementary file 1 — Additional file 1: Appendix 1. Suggestions about comprehensibility, relevance, and comprehensiveness. [file 40359_2024_1557_MOESM1_ESM.docx]

**Appendix 1. Suggestions about comprehensibility, relevance, and comprehensiveness**

|  | Category | Description |
| --- | --- | --- |
| Comprehensibility | **Difficulty in discriminating the meaning of “まれに (rarely)” and “しばしば (often)”** | **The word “まれに (rarely)” bothered me. I guess you mean sometimes?** |
|  |  | **What is the difference between rarely and often?** |
|  |  | **I almost confused the word rarely with often.** |
|  |  | **The expression “しばしば (often)” is confusing.** |
|  |  | **I don't know the definition of often.** |
|  | No concrete suggestions | A little confusing. |
|  |  | Difficult to understand. |
|  |  | It is difficult to name an alternative that is easier to understand. But I feel there is room for something creative. |
|  |  | There was a problem that was difficult to understand. |
|  |  | A little confusing. |
|  |  | It was a little confusing. |
|  | Suggestions by one participant | Evaluating using a number scale is easier. |
|  |  | I understood, but it could be more specific. |
|  |  | The expression, I just couldn't do it; it was difficult. |
| Relevance | No concrete suggestions | Not much of a match. |
|  |  | Irrelevant. |
|  | No suggestions regarding bedtime procrastination or activities during bedtime procrastination | Sometimes after looking at my cell phone, I can't fall asleep. |
|  |  | I fiddle with my phone. |
|  | Suggestions by one participant | I don't want to go to work. |
|  |  | Because I never finish what I have to do. |
|  |  | I tend to procrastinate. |
|  |  | If you have something on your mind, you can't sleep, right? |
| Comprehensiveness | No concrete suggestions | I don't think so. |
|  |  | I don't think I've covered it all, but I can't go so far as to give specific examples. |
|  | Irrelevant suggestions  (Activities during bedtime procrastination) | Sometimes I look at my cell phone and can't sleep. |
|  |  | I check my phone and end up going to bed late. |
|  |  | I fiddle with my phone. |
|  |  | I try to go to bed early, but it is always late when I realize it because of my phone or TV. |
|  | No suggestions regarding bedtime procrastination (daily functions) | Because there is no item that responds to health problems such as frequent urination at night, and to me, this problem is a sleep problem. |
|  |  | Daytime activities can affect sleep but they are not mentioned. |
|  |  | I thought it would be good to have a section on mental or physical fatigue. |
|  | No suggestions regarding bedtime procrastination  (reasons for bedtime procrastination) | For example, the body feels sleepy, but the mind is against the will to fall asleep. |
|  |  | Sleeping seems like a waste of time. |
|  |  | It is a waste of time to sleep. |
|  |  | I want to get enough sleep and I understand the effects on my body but I want something other than sleep, time to “relax” by looking at social networking sites or reading manga, and then I become sleepy, but I can't start preparing for bed. |
|  |  | I have to do household chores, and time passes quickly. I go to bed late. |
|  | Suggestions by one participant | I get distracted by other things and my eyes glaze over. |

Bold = suggestions reflected in response to the BPS-J.
