# Supplementary material for: Development and validation of the Japanese version of the Bedtime Procrastination Scale (BPS-J)
Source: BMC Psychol. 2024 Feb 1;12:56. doi: 10.1186/s40359-024-01557-4 (PMC10832274; doi:10.1186/s40359-024-01557-4)
Supplement: Supplementary file 2 — Additional file 2: Appendix 2. Translation of Instructions and items of the Bedtime Procrastination Scale. [file 40359_2024_1557_MOESM2_ESM.docx]

**Appendix 2. Translation of Instructions and items of the Bedtime Procrastination Scale**

| **The Bedtime Procrastination Scale English version**  **developed by Kroese et al(15)** | **The Bedtime Procrastination Scale Japanese version** |
| --- | --- |
| For each of the following statements, please decide whether it applies to you using a scale from 1 (*almost*) *never* to 5 *(almost) always*. | 以下の各項目について、あなたに当てはまるかどうか、1（ほとんどない）から5（ほとんどいつもある）までの数字でお答えください。 |
|  |  |
| 1. I go to bed later than I had intended. | 1.自分が意図していたより、寝る時間が遅くなる。 |
| 1. I go to bed early if I have to get up early in the morning (R). |  |
| 1. If it is time to turn off the light at night I do it immediately (R). | 3. 夜、明かりを消さなければならない時間になったら、すぐに消す。(R) |
| 1. Often I am still doing other things when it is time to go to bed. | 4. 寝る時間になっても、他のことをしていることが多い。 |
| 1. I easily get distracted by things when I actually would like to go to bed. | 5. 本当は寝ようと思っている時でも、すぐに他の事に気をとられてしまう。 |
| 1. I do not go to bed on time. | 6. 決まった時間に寝ない。 |
| 1. I have a regular bedtime which I keep to (R). | 7. 決まった就寝時間があり、それを守っている。(R) |
| 1. I want to go to bed on time but I just don’t. | 8. 決まった時間に寝たいと思うが、できない。 |
| 1. I can easily stop with my activities when it is time to go to bed (R). | 9. 寝る時間になったら、それまで行っていた活動を容易に止められる。(R). |

Item 2 “翌朝早く起きなければならないときは、早く寝る。(I go to bed early if I have to get up early in the morning.)” was eliminated from the scale based on the results of the structural validity.

(R) indicates that the question requires reverse scoring.
